# Self-Reported Complaints as Prognostic Markers for Outcome After Mild Traumatic Brain Injury in Elderly: A Machine Learning Approach

**DOI:** 10.3389/fneur.2021.751539

**Published:** 2021-12-02

**Authors:** Mayra Bittencourt, Sebastián A. Balart-Sánchez, Natasha M. Maurits, Joukje van der Naalt

**Affiliations:** Department of Neurology, University Medical Center Groningen, University of Groningen, Groningen, Netherlands

**Keywords:** mild traumatic brain injury (mTBI), older age, post-traumatic complaints, post-concussive symptoms, prognosis, recovery, machine learning

## Abstract

Self-reported complaints are common after mild traumatic brain injury (mTBI). Particularly in the elderly with mTBI, the pre-injury status might play a relevant role in the recovery process. In most mTBI studies, however, pre-injury complaints are neither analyzed nor are the elderly included. Here, we aimed to identify which individual pre- and post-injury complaints are potential prognostic markers for incomplete recovery (IR) in elderly patients who sustained an mTBI. Since patients report many complaints across several domains that are strongly related, we used an interpretable machine learning (ML) approach to robustly deal with correlated predictors and boost classification performance. Pre- and post-injury levels of 20 individual complaints, as self-reported in the acute phase, were analyzed. We used data from two independent studies separately: UPFRONT study was used for training and validation and ReCONNECT study for independent testing. Functional outcome was assessed with the Glasgow Outcome Scale Extended (GOSE). We dichotomized functional outcome into complete recovery (CR; GOSE = 8) and IR (GOSE ≤ 7). In total 148 elderly with mTBI (median age: 67 years, interquartile range [IQR]: 9 years; UPFRONT: *N* = 115; ReCONNECT: *N* = 33) were included in this study. IR was observed in 74 (50%) patients. The classification model (IR vs. CR) achieved a good performance (the area under the receiver operating characteristic curve [ROC-AUC] = 0.80; 95% CI: 0.74–0.86) based on a subset of only 8 out of 40 pre- and post-injury complaints. We identified increased neck pain (*p* = 0.001) from pre- to post-injury as the strongest predictor of IR, followed by increased irritability (*p* = 0.011) and increased forgetfulness (*p* = 0.035) from pre- to post-injury. Our findings indicate that a subset of pre- and post-injury physical, emotional, and cognitive complaints has predictive value for determining long-term functional outcomes in elderly patients with mTBI. Particularly, post-injury neck pain, irritability, and forgetfulness scores were associated with IR and should be assessed early. The application of an ML approach holds promise for application in self-reported questionnaires to predict outcomes after mTBI.

## Introduction

Traumatic brain injury (TBI) is one of the most important causes of morbidity and mortality in adults (1). Mild TBI (mTBI) accounts for 85% of the cases (2, 3). Most patients who sustained mTBI report post-injury complaints within the first weeks after injury, which can involve cognitive (e.g., forgetfulness and poor concentration), emotional (e.g., irritability and anxiety), and/or physical domains (e.g., headaches and fatigue). Although post-injury complaints usually resolve within 3 months after injury, they may persist for months to years in a subgroup (≈20%), which is often referred to as post-concussion syndrome (PCS) (4). According to the International Classification of Diseases (ICD)-10 definition, the diagnosis of PCS requires the presence of three or more post-injury complaints for at least 4 weeks (5). However, individual complaints are non-specific to PCS and can also be found in healthy individuals and across a variety of clinical populations (6–8).

Post-injury complaints are frequently assessed through self-reported questionnaires that quantify the relative severity of the current complaints compared to the pre-injury level (9–12). Thus, pre-injury status must be recalled by the patients when filling in the questionnaires and their subjective perception can affect the rating of self-reported complaints (13). It has been suggested that individuals might tend to underestimate past problems after a traumatic event, such as sustaining an mTBI, which is referred to as “good-old-days” bias (14). In the elderly, additionally, a higher rate of pre- or co-morbid complaints is expected (15). Nevertheless, few studies have investigated the effects of pre-injury complaints on long-term disability after mTBI (16), and particularly information concerning the elderly population is lacking (15).

Increasing age has been identified as an independent predictor of worse outcomes after mTBI (17). As the world population continues to age, the number of elderly sustaining mTBI will further increase. The elderly with mTBI are more likely to suffer from comorbidities than their younger counterparts and constitute a vulnerable group, which is more prone to develop PCS (15, 18). Therefore, the geriatric population has been defined as a priority research area for mTBI prognostic studies (19, 20). Nonetheless, few studies have investigated the prognosis of geriatric mTBI so far (1, 15, 21, 22).

Identifying prognostic markers of incomplete recovery (IR) can assist clinicians in offering optimal, personalized clinical care for those patients at risk (1, 16). Injury-related functional impairments can be assessed using the Glasgow Outcome Scale Extended (GOSE), which is the most used outcome measure in TBI studies (23, 24). This scale is often combined with the number and severity of post-injury complaints in the (sub)acute phase after mTBI to indicate IR (25, 26). In mTBI research, the lack of specificity of post-injury complaints and the presence of comorbidities, particularly, in the elderly are examples of problems that hamper prognostic studies. Machine learning (ML) algorithms are powerful tools for pattern recognition that are increasingly used due to their capacity to deal with issues present in complex data, such as a high number of (potentially dependent) predictors and non-linear relationships in the data, that more classical statistical approaches, such as (logistic) regression, are not well-suited for. In the medical field, ML techniques can be applied for identifying risk factors and for developing outcome prediction models (27–29).

In this study, we aimed to identify which individual (pre- and post-injury) complaints are potential prognostic markers for long-term functional impairments (i.e., IR) in elderly patients with mTBI. To achieve this aim, we used an interpretable ML-based approach employing a support vector machine (SVM).

## Methods

### Study Population

Data were obtained as part of two independent larger prospective follow-up studies. Both were conducted at a university level 1 trauma center. Patients with mTBI were consecutively included at the emergency department (ED) between March 2013 and February 2015 for the UPFRONT study and between November 2018 and September 2019 for the ReCONNECT study. The diagnosis of mTBI was based on the following criteria: attending the hospital with an mTBI defined by a Glasgow Coma Scale (GCS) score of 13–15, loss of consciousness ≤ 30 min, and/or post-traumatic amnesia of ≤ 24 h (30). For both studies, comprehension of the Dutch language was necessary. The age of inclusion for the UPFRONT study was 16 years or older and for the RECONNECT study, 60 years or older. Here, we only analyzed data from participants aged 60 years or older for whom the 6-month outcome assessment (i.e., GOSE score) was available, and missing data for predictors were no higher than 5% (i.e., maximum 2 out of 40 questions left unanswered). Exclusion criteria were a history of drug or alcohol abuse, and/or a major psychiatric or neurologic disorder as identified by the attending or ward physician. Patients without a permanent home address were also excluded. Both studies were approved by the local Medical Ethics Committee, and written informed consent was obtained from all participants. All procedures were performed according to the declaration of Helsinki (2013).

### Measurements

#### Complaints

The Head Injury Symptoms Checklist (HISC) is a 21-item post-traumatic questionnaire (9), derived from the Rivermead Post-concussion Symptoms Questionnaire (RPQ) (10). Patients are asked to score each complaint both on the pre-injury level and on the current level with values ranging from 0 to 2 (never = 0, sometimes = 1, and often = 2). For each complaint, the post-injury score is calculated by subtracting the pre-injury score from the current score. In this manner, complaints that were new or increased in severity after mTBI could be identified. Both post-injury scores (i.e., current complaints scores corrected for pre-injury level) and pre-injury scores were used as features for classification. The HISC was administered to patients within the first 2 weeks after injury. One of the 21 symptoms (intolerance to alcohol) was excluded from the analysis as patients are usually refraining from alcohol consumption in the (sub)acute phase after injury.

#### Outcome

The GOSE measures functional outcome by TBI-related changes relative to pre-injury abilities on a scale that ranges from 1 (death) to 8 (complete recovery, CR). Questions, where there has been no change in comparison to pre-injury functioning status, were ignored for the definitive scoring in accordance with the manual (31). The outcome was dichotomized into CR (GOSE = 8) and IR (GOSE ≤ 7) (32). The GOSE was administered 6 months after the injury for both UPFRONT- and ReCONNECT studies. Previous studies indicate that functional outcome stabilizes around 3 months after injury. At 6 months, further improvements in the functional status are therefore unlikely for mTBI (33).

### ML Approach

Predicting the outcome of a patient with mTBI (i.e., distinguishing between CR and IR) can be considered a binary classification problem.

In the context of ML, SVMs provide one of the commonly used supervised learning algorithms that perform well on binary classification problems and minimize overfitting (34). In supervised methods, the model is trained using input data with known labels that are provided by the user.

In this study, we designed an SVM-based classification model to separate patients with IR and patients with CR (class labels defined as 1 and −1, respectively).

Although SVM is a well-established ML method, employing it to determine prognostic markers based on self-reported complaints is, to the best of our knowledge, a novel approach. Therefore, to validate our approach, we performed a preliminary analysis on a similar, but supposedly simpler, classification problem, which consisted of discriminating patients in the (sub)acute phase after injury from healthy controls based on self-reported complaints. The details of this preliminary analysis are presented in the Supplementary Material.

### Data Preparation

Classification features: The feature vector ***x***(k), where k is the total number of features, consisted of the 20 pre-injury and the 20 post-injury scores measured with the HISC questionnaire, thus *k* = 40. The rows of the classification feature matrix ***X***(m, k) consisted of the feature vectors from all observations, where m is the total number of participants. The class labels vector ***y***(m) contained the class for each feature vector in ***X*** (CR or IR).

Cross-validation training, external validation, and independent testing subsets: Data of the UPFRONT study were used for cross-validation training and external validation. The cross-validation training and the external validation subsets consisted of a random, stratified selection of 80 and 20% of the UPFRONT study dataset (35), respectively, and were obtained by using the function “cvpartition” (MATLAB 2020). The external validation dataset was held out from the cross-validation training process and left unseen until external validation. All data from the ReCONNECT study dataset were used for independent testing (i.e., confirmatory analysis) and were left unseen during the cross-validation training and external validation. The datasets from ReCONNECT and UPFRONT studies were not combined to avoid adding heterogeneity to the training dataset, which could reduce the efficiency of the feature selection process.

### Model Training and Features Selection

We built a script based on an SVM training algorithm (“fitcsvm,” MATLAB 2020) and a recursive backward feature selection procedure. The SVM algorithm returns trained SVM classifiers for binary classification based on a matrix with data from predictive features (***X***) and a vector (***y***) with the associated known labels. ***X*** and ***y*** were used as input to the SVM training algorithm using a linear kernel function. Hyperparameters were defined as: kernel scale (default, 1) and Box Constraint (default, 1).

A recursive backward selection method was used for the subset selection of features. The order of the features (complaints) followed the same order as they are listed in the original questionnaire. At the first step, all features (individual complaint scores as assessed by the HISC questionnaire) were selected. At each subsequent step, the number of features was reduced by one. Additionally, at each subsequent step, all observations were initially included and observations with missing data (coded as “NaN”; Not a Number) for any of the selected features were (stepwise) identified and excluded from the dataset to maximize data usage per participant. The feature to be removed is the one that results in the smallest loss of classification performance (as measured by average the area under the receiver operating characteristic curve [ROC-AUC]) when removed. The average ROC-AUC for a given subset was calculated based on r-repeated k-fold cross-validation (*r* = 10, *k* = 5) (35). In the case of multiple occurrences with an identical result, the first occurrence was selected. The procedure was repeated until only two features were left. The selected (locally) optimal number of features correspond to the smallest number of features that attained the maximum average classification performance (i.e., maximum average ROC-AUC based on 10-repeated 5-fold cross-validation). The selected (locally) optimal subset of features corresponds to the subset with the selected number of features that attained the maximum classification performance (i.e., maximum ROC-AUC based on 10-repeated 5-fold cross-validation).

After the feature selection process, the final model was trained using the optimal subset of features. This approach reduces the risk of overfitting.

### Statistical Analysis and Performance Assessment

We verified whether there were any differences between (UPFRONT and ReCONNECT) datasets in age and 6-month outcome by independent-samples median tests and Chi-square tests, respectively (as variables were non-normally distributed). Additionally, we verified whether there were any differences in age between groups (CR and IR), per dataset, by independent-samples median tests.

For the final model, we calculated the ROC-AUC to assess model performance (36). Secondary performance measures were sensitivity, specificity, F1-score, and total accuracy [please refer to Sokolova et al. (37) for details about their calculation]. For the feature weights and the performance measures (ROC-AUC, sensitivity, specificity, and total accuracy), mean and 95% CIs were calculated based on 5-fold cross-validation (35).

Statistical significance of mean weights being different from the statistical distribution under the null hypothesis was calculated using permutation tests (38, 39). For 10,000 permutations, new models were built after randomly permuting class labels to empirically estimate the statistical distribution of the weights under the null hypothesis. The *p*-values were calculated as the proportion in the distribution under the null hypothesis that is greater than or equal to the absolute value of the mean weight value obtained by using the original (non-permutated) data.

## Results

### Participant Characteristics

In total, 148 elderly with mTBI were included in this study (median age: 67 years, IQR: 9 years; UPFRONT: *n* = 115; ReCONNECT: *n* = 33).

The summary of characteristics per (UPFRONT and ReCONNECT) dataset of patients with mTBI and for all patients (both datasets combined) is presented in Table 1. Independent-samples median tests neither indicated any significant difference in age between datasets nor between groups (IR vs. CR) as assessed per dataset.

**Table 1:** Patient characteristics per dataset.

	**All patients**	**UPFRONT dataset**	**ReCONNECT dataset**	***p*-value**
	**(Combined datasets, *N* = 148)**	**(Cross- and external-validation, *N* = 115)**	**(Independent Testing, *N* = 33)**	
Age (years), Median (IQR)	67 (9)	66 (9)	70 (9)	0.057^a^
Outcome (IR/CR)	74/74	55/60	19/14	0.323^b^

a *An independent-samples median test*.

b *Chi-square test*.

*CR, complete recovery; IR, incomplete recovery*.

**Figure 1:**
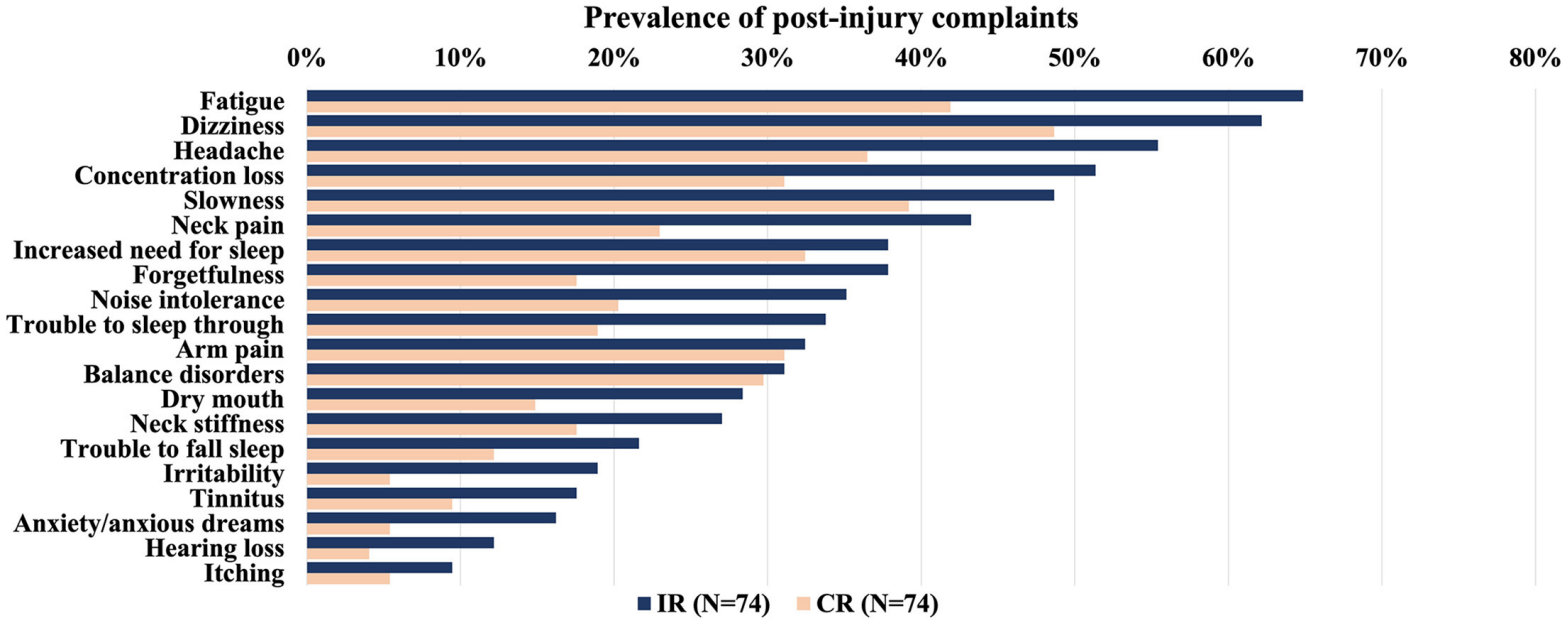
Overview of the prevalence of self-reported post-injury complaints (%) in patients with mTBI (UPFRONT and ReCONNECT datasets) within 2 weeks after injury per outcome group (IR: incomplete recovery or CR: complete recovery). Complaints ordered by prevalence in the IR group from highest to lowest. There were no differences between IR and CR groups (Mann-Whitney *U* test, *p* <0·05, Bonferroni corrected for multiple comparisons). mTBI, mild traumatic brain injury.

**Figure 2:**
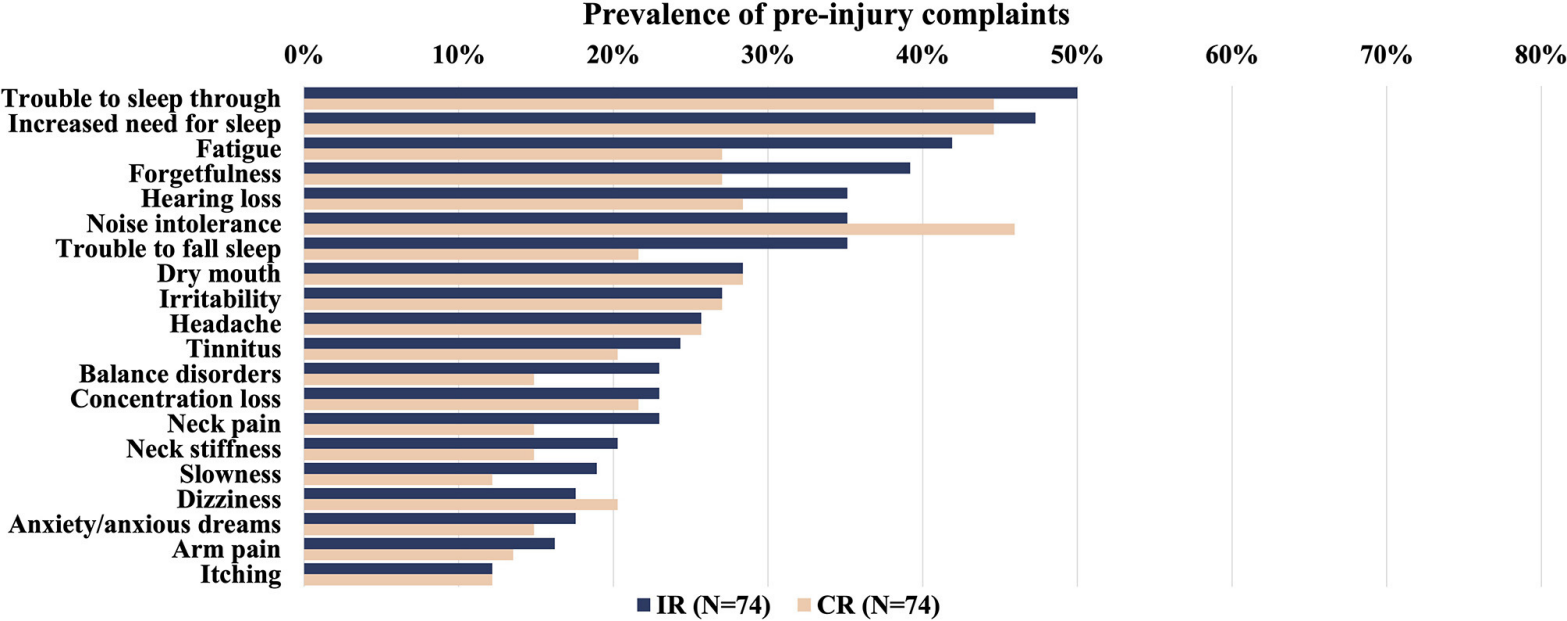
Overview of the prevalence of self-reported pre-injury complaints (%) in patients with mTBI within 2 weeks after injury per outcome group (IR: incomplete recovery or CR: complete recovery). Complaints ordered by prevalence in the IR group from highest to lowest. There were no differences between groups (Mann Whitney *U* test, *p* < 0.05, Bonferroni corrected for multiple comparisons). mTBI, mild traumatic brain injury.

The prevalence of post-injury complaints per group (CR or IR) for both datasets combined is presented in Figure 1. Additionally, the prevalence of pre-injury complaints per group (CR or IR) is presented in Figure 2.

### Feature Selection and Performance Assessment

**Figure 3:**
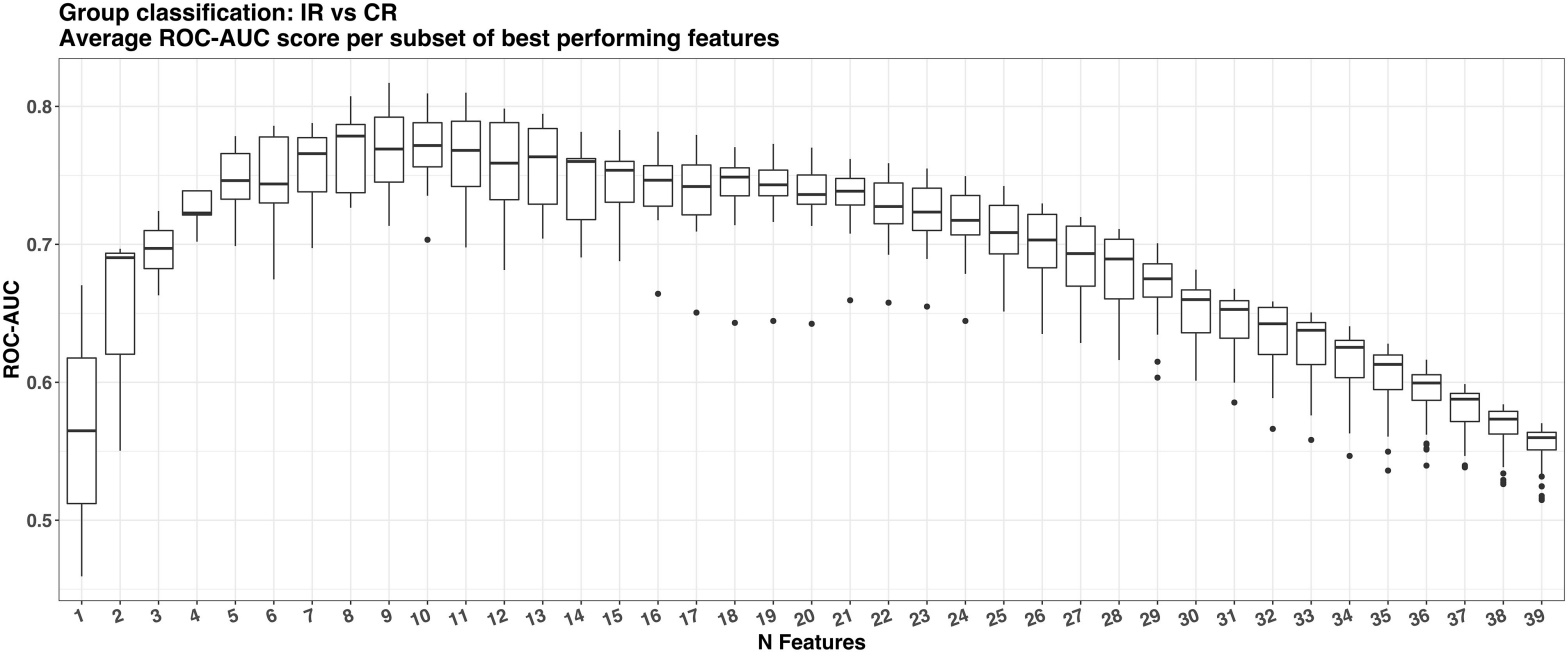
Boxplots of the average area under the receiver operating characteristic curve (ROC-AUC) per number of features in the subset during the feature selection stage using the cross-validation dataset.

The ML feature selection procedure showed that the maximum average ROC-AUC of 0.77 was achieved with 8 out of the 40 available features. Subsequently, the best model with eight features was determined that achieve the maximum ROC-AUC of 0.81 (see Figure 3).

The selected subset containing eight features for prediction, ranked by order of exclusion, is detailed in Table 2. The ML feature selection procedure identified 3 out of 40 assessed pre- and post-injury complaint scores as significant predictors of IR, namely, post-injury neck pain score (weight: 1.38 [95% CI: 1.09, 1.68], *p* = 0.001), post-injury irritability score (weight: 1.91 [95% CI: 1.61, 2.21], *p* = 0.011), and post-injury forgetfulness score (weight: 1.28 [95% CI: 1.02, 1.54], *p* = 0.035) as shown in Table 2. Additionally, post-injury arm pain (Weight: −1.02 [95% CI: −1.15, −0.88], *p* = 0.021) was the only complaint score identified as a significant predictor of CR.

**Table 2:** Optimal subset of features for prediction ranked by order of exclusion if selection process continued until the last feature (1: last feature to be excluded).

**Features**	**Pre-/Post-injury score**	**Selection ranking**	**Weight, mean, 95% CI [ LL, UL]^a^**	***p*-value^b^**
Neck pain	Post	1	1.39 [1.09, 1.68]	0.001
Arm pain	Post	2	−1.02 [−1.15, −0.88]	0.021
Irritability	Post	3	1.91 [1.62, 2.21]	0.011
Forgetfulness	Post	4	1.28 [1.02, 1.54]	0.035
	Pre	5	0.70 [0.42,0.97]	0.114
Slowness	Post	6	−0.73 [−0.84, −0.63]	0.092
Headache	Pre	7	−0.63 [−0.87, −0.39]	0.143
Increased need for sleep	Pre	8	0.37 [0.18, 0.57]	0.193

a *95% CI values calculated based on 5-fold cross-validation; LL, lower level; UL, upper level*.

b *Statistical significance based on permutation tests (N models built with random class labels permutations, N = 10,000)*.

*Blue: significant features with positive weight, contribute to incomplete recovery (IR) prediction; pink: significant features with negative weight, contribute to complete recovery (CR) prediction*.

**Figure 4:**
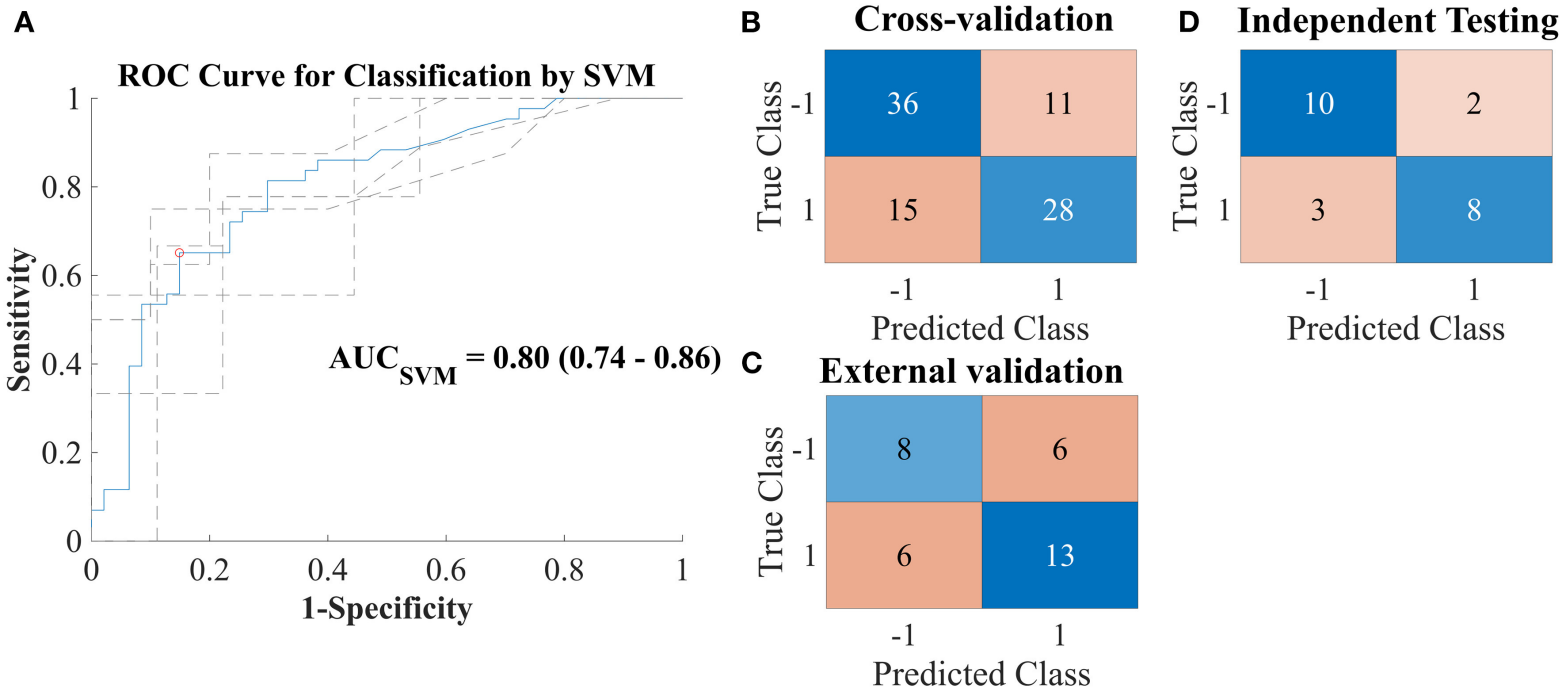
**(A)** Receiver operating characteristic curve for prediction of group classification—complete recovery (CR; class label: 1) vs. incomplete recovery (IR; class label: −1)—based on a linear support vector machine (SVM) model built with the optimal subselection of discriminating features and 5-fold cross-validation. Dashed lines indicate results per fold. **(B–D)** Confusion matrices for the predictions of the SVM model vs. true labels on cross-validation dataset [**(B)**; 80% of the UPFRONT dataset], external validation on unseen data [**(C)**; 20% of the UPFRONT dataset], and testing on an independent dataset [**(D)**; ReCONNECT dataset].

The final trained, cross-validated model yielded an average ROC-AUC of 0.80 [95% CI: 0.74, 0.86] (see Figure 4A). The confusion matrices displaying the classification results for the cross-validation training, for the external validation, and the independent testing are also shown in Figures 4B–D.

Secondary performance measures are detailed in Table 3. The classifier achieved slightly higher performance for sensitivity in the external validation (Sensitivity_EV_ = 0.73) and in the independent testing (Sensitivity_IT_ = 0.68) than in the cross-validation training (Sensitivity_CV_ = 0.65 [95% CI 0.46, 0.83]). The performance for specificity, however, was lower in the independent testing (Specificity_IT_ = 0.57) than in the cross-validation training (Specificity_CV_ = 0.76 [95% CI 0.61, 0.92]) and in the external validation (Specificity_EV_ = 0.78).

**Table 3:** Performance of selected predictive support vector machine (SVM) model on cross-validation dataset and external validation on an independent dataset.

	**mTBI CR vs. IR**		
	**Cross-Validation**	**External**	**Independent**
		**validation**	**testing**
Sensitivity	0.65 (0.46–0.83)	0.73	0.68
Specificity	0.76 (0.61–0.92)	0.83	0.57
Total Accuracy	0.71 (0.68–0.74)	0.78	0.64
F1-score	0.68 (0.64–0.72)	0.78	0.62
ROC-AUC	0.80 (0.74–0.86)	-	-

*mTBI, mild traumatic brain injury; CR, complete recovery; IR, incomplete recovery; ROC-AUC, area under the receiver operating characteristic curve*.

## Discussion

The main goal of this study was to identify which individual pre- and post-injury complaints in the elderly during the (sub)acute phase after mTBI are the strongest predictors (i.e., potential prognostic markers) for IR. We used an ML approach employing an SVM to robustly deal with correlated predictors, achieve high classification performance and optimal interpretability of results. This methodology allowed us to assess how different complaints relate to functional outcomes after mTBI.

The classification model achieved a good performance (ROC-AUC 0.80) based on a subset of 8 (out of 40) self-reported pre- and post-injury complaints. From this subset, three post-injury scores (which are corrected for pre-injury level) were identified as significant predictors for IR and one as a significant predictor for CR, whereas none of the pre-injury scores were identified as significant predictors for IR/CR, independently. We will detail and discuss our findings below.

Our results indicated post-injury neck pain, irritability, and forgetfulness scores as the most relevant predictors for IR in the elderly population. Importantly, these three aforementioned scores represent post-injury complaints that are part of, respectively, physical, emotional, and cognitive domains, corroborating with the notion that the involvement of a higher number of domains among post-injury complaints makes PCS (4, 5) and thus IR more likely.

In comparison to the cross-validation, the external validation achieved similar sensitivity and specificity levels, indicating that the model generalized for unseen data. The independent testing achieved similar sensitivity levels, but lower specificity levels, meaning that predictors of CR did not generalize well to a different population and should be interpreted with additional caution. Perhaps complaints among patients that recover well are less homogeneous than complaints among patients with IR. To the goal of this study (i.e., identifying potential prognostic markers for elderly patients at risk of IR), sensitivity is more important than specificity, however. In other words, for this study, missing a patient at risk of IR would be more costly than mistakenly classifying a patient that will probably recover well as being at risk of IR. Therefore, we will focus on discussing the predictors of IR.

Concerning the role of cognitive impairments in the recovery process in older adults after mTBI, we found that the self-assessment of increased forgetfulness after injury entails prognostic value for determining IR. Currently, there is increased awareness of a possible association between sustaining an mTBI at an older age and the early onset of neurodegenerative diseases, such as mild cognitive impairment and dementia (40–42). Although this possible association suggests that mTBI may act as a stressor in the normal aging process (42, 43), a clear mechanism relating (pre- and post-injury) cognitive complaints to functional outcome after mTBI has not been established. Here, the possibility that (some of the) patients were facing pre-existing memory deficits due to reasons that are unrelated to mTBI and/or misattributed increased forgetfulness to the injury cannot be ruled out, although patients with either pre-diagnosed or suspected neurodegenerative diseases were not included in this study. Moreover, it is known that several older patients with mTBI are able to cope with pre- and post-injury cognitive impairments and recover well (15). Perhaps our and previous results could be elucidated by the theoretical concept of cognitive reserve, which attempts to explain individual differences in cognitive functioning after brain damage (44). According to this theoretical framework, the elderly might cope with brain damage without clinical manifestation as long as cognitive reserve allows them to maintain cognitive performance (44, 45). We encourage future studies to disentangle the mechanisms behind cognitive functioning/decline, the development of cognitive complaints, and the role of cognitive reserve in the recovery process after mTBI at the older age.

With regard to the emotional domain, we found a post-injury irritability score as a predictor of IR after sustaining an mTBI at an older age. Previous studies on adults with mTBI identified irritability among post-traumatic complaints that can persist for longer than 1 year (46, 47) and are associated with its severity (48). Interestingly, Yang et al. (49) studied adults with mild- and moderate-to-severe TBI and found that annoyance (i.e., an aspect of irritability) was self-reported by patients with mTBI but remained unnoticed by their caregivers. Moreover, post-injury irritability might be associated with impaired cognitive capabilities, such as information processing in adults after mTBI (49). Our results add to evidence that both increases in irritability and forgetfulness from pre- to post-injury (as self-reported in the subacute phase) indicate an increased risk for worse outcomes in the elderly mTBI population. Therefore, the early assessment of subjective complaints regarding alterations in cognitive capabilities and their interaction warrants further investigation.

Regarding the role of physical impairments in the recovery process, we identified the post-injury neck pain score as a predictor for IR in elderly patients. Previous studies suggested that acceleration/deceleration of the head during mTBI can cause concomitant damage to cervical spine tissues, which might be reflected by (acute) neck pain (50). Further, previous studies indicated that a possible interplay (between mTBI and concomitant cervical involvement) might lead to increased risk for the development of PCS (50–52) and/or incomplete functional recovery in patients with mTBI. Our findings suggest that cervical involvement in the injury might be a risk factor for IR in elderly patients, as well.

Additionally, we identified post-injury arm pain score as a predictor for CR. Perhaps pain (outside neck/head region) during the subacute phase after an injury is found across patients regardless of their outcome, reflecting merely contusional lesions to muscles and joints that mostly resolve well over time and, in the absence of (a combination of) post-injury neck pain and/or emotional and cognitive complaints, do not affect the course of recovery (53**?**). However, the prediction of CR did not generalize well to the independent dataset and we therefore refrain from further interpreting this result.

Overall, our findings suggest an interplay between physical, emotional, and cognitive limitations interfering with long-term recovery in elderly with mTBI. Notably, the most predictive factors for IR (i.e., forgetfulness, irritability, and neck pain) were not the most prevalent (i.e., fatigue, headache, and dizziness). As such, certain somatic complaints might be typical, perhaps transient complaints that are found in the early phase after mTBI (at an older age), with reduced value for determining long-term outcome. Another interesting finding is that not a high number but a selection of complaints can yield more prognostic information.

Clinical implications of the aforementioned findings include the potential of improving the identification of the subgroup of older adults at risk of experiencing an IR trajectory after mTBI. Additionally, information from questionnaires could be used more efficiently, either by reducing the number of questions to be asked or by increasing interpretability (e.g., developing additional (sub)scores, according to the purpose of the assessment). Nevertheless, the mechanisms underlying this interaction between older age and mTBI sequelae, comprising cognitive, emotional, and physical complaints, among other factors, are still largely unknown. Although the self-assessment of complaints can provide valuable and predictive information, the possibility that (some of the) patients might misattribute complaints as being new or having their severity increased after mTBI exists, which hampers the identification of mTBI sequelae at an individual level. Future studies are encouraged to assess a selection of individual self-reported complaints and include clinically diagnosed pre-injury factors and/or mTBI symptoms in their analysis to advance the knowledge in the field.

Moreover, we identified four post-injury scores (which are controlled for pre-injury complaints status) as significant predictors for IR/CR, whereas none of the pre-injury scores were separately identified as significant predictors. Therefore, the subjective assessment of complaints as being new or having their severity increased after mTBI (which requires pre-injury assessment) has a predictive value, although the subjective assessment of pre-injury complaints (independently) did not yield predictive information, possibly due to inaccuracies. Of note, the HISC questionnaire measures the (pre- and post-injury) severity of a list of commonly occurring complaints after TBI and is not designed to provide a complete clinical assessment of pre-injury status. Therefore, other pre-injury factors (e.g., physical activity level, social engagement, and comorbidities under medical treatment or not) were not analyzed in this study but could potentially be predictors for IR/CR, as well.

Further, our outcome classification model (IR vs. CR) achieved a good performance (ROC-AUC 0.80) based on only a subset of self-reported complaints. Nonetheless, the performance of our classifier is comparable or even superior to previous predictive models that were built based on a broad variety of predictors (e.g., clinical characteristics, injury mechanism, and among others) (54), suggesting a benefit in applying our ML approach. We chose to focus solely on self-reported complaints to explore this specific aspect of the mTBI clinical assessment in elderly patients. At an older age, complaints can widely vary in number and severity both pre- and post-injury making them difficult to be processed and disentangled. Therefore, if prognostic markers can be identified from questionnaires, the burden for the clinicians when assessing large amounts of patient information to yield information for prognosis could be reduced by an ML approach. Possibly our findings can improve the early identification of the subgroup of older patients at risk of IR after mTBI, increase the usefulness of questionnaires, and lead to new insights toward personalized treatment.

### Methodological Considerations, Strengths, and Limitations

In our study, we opted for a linear SVM approach combined with backward feature selection. In comparison to classical statistical approaches, this method deals adequately with variables that (highly) correlate with each other (i.e., multicollinearity) and is less affected by the presence of outliers in the data. The application of ML for the analysis of individual complaints after mTBI is, to the best of our knowledge, a new approach. Therefore, to explore the validity of this ML approach, we performed a preliminary analysis on a similar, presumably simpler, classification problem (patients with mTBI vs. healthy controls; see Supplementary Material). In the preliminary analysis, we achieved an excellent performance (ROC-AUC 0.91) based on a relatively small set (9 out of 20) of self-reported complaints. Although the results of this preliminary analysis have low clinical value (clinicians can already distinguish a patient with mTBI from a healthy control), this step allowed us to confirm the validity of the proposed approach.

Additionally, the linear SVM variant allows reasonably straightforward interpretability, which is highly desirable in exploratory analysis and for the translation of results to the clinical setting. However, although linear SVM can robustly deal with outliers and some level of non-linearity, linear methods are most likely suboptimal if dealing with heterogeneous datasets, which is frequently the case when analyzing clinical populations (such as an mTBI population).

Regarding the optimization procedure, which is performed to increase performance by selecting the most important features for classification, the following should be taken into consideration. This procedure discards not solely the features with poor predictive power, but also those features that are highly correlated to the selected ones.

Some limitations to this study should be mentioned. First, self-reported complaints are one of several aspects that determine outcome after mTBI. While focusing on self-reported complaints allowed to gain knowledge on this aspect of outcome determination, complaints are more likely to be associated with IR than with CR. Therefore, the identification and inclusion of prognostic factors to the model that is likely to be associated with CR (e.g., higher education level) would possibly lead to higher classification performance.

Second, as previously mentioned, self-reported information entails subjectivity and does not provide a complete assessment of pre- and post-injury status. It is possible that (some of the) patients have other comorbidities interfering with the recovery trajectory, have misattributed complaints to the mTBI, and/or have developed complaints about reasons that are unrelated to the mTBI. Therefore, it is not possible to demonstrate causal relationships between mTBI and any (combination of) the assessed complaints. Nonetheless, self-reported complaints are easy to obtain and, frequently, are one of a few sources of information about the pre-injury status. With the present study, we demonstrate that self-reported complaints, despite the subjectivity involved, entail prognostic value for IR after mTBI at the older age. Future studies are encouraged to investigate underlying mechanisms to explain a possible association of post-traumatic complaints with outcomes.

Third, factors that are either indirectly related or unrelated to the brain injury itself might partly contribute to long-term IR. As an example, it has been previously identified that psychological factors (i.e., emotional distress and maladaptive coping experienced early after injury) in combination with pre-injury mental health problems, education, and age are important predictors for recovery in adult mTBI populations (32). In the elderly, more complex interactions between mTBI sequelae, (pre-existing) factors, and/or co-occurring injuries are likely to play a role in the recovery process. Hence, the identification of functional impairments that are specific to mTBI sequelae becomes even more challenging in this group, which might in fact render the term post-concussive syndrome inapplicable for this population. In sum, it is possible that some of the patients with IR (i.e., GOSE ≤ 7), while being fully recovered from any neurological sequelae from mTBI, were still experiencing functional impairments at 6 months after injury due to other pre-existing factors or co-occurring injuries.

Finally, although we performed testing using an independent dataset to verify the generalizability of the model, our sample size was relatively small. Future confirmatory studies with the use of this ML approach are encouraged both to identify new individual prognostic factors (e.g., injury characteristics and personality factors) that could increase the performance of outcome prediction models and to verify the potential of the identified prognostic markers for generalizing to other populations.

## Conclusion

A subset of individual pre- and post-injury complaints self-reported in the (sub)acute phase after mTBI can predict long-term functional outcomes in the elderly population. ML is a valuable approach for the identification of potential prognostic markers. The early assessment of physical complaints that indicate cervical spine involvement, subjective irritability, and (both pre- and post-injury) memory functioning impairments might facilitate the identification of elderly at risk of poor outcome, improving the rehabilitation process. Further studies are needed to confirm the validity of the identified self-reported complaints as prognostic markers and to clarify the mechanisms that associate them with the recovery process after mTBI.

## Data Availability Statement

The datasets presented in this article are not readily available because patients did not consent to sharing data for purposes other than the current study. Requests to access the datasets should be directed to Mayra Bittencourt, m.bittencourt.villalpando@umcg.nl.

## Ethics Statement

The studies involving human participants were reviewed and approved by Medical Ethical Committee of the University Medical Center Groningen. The patients/participants provided their written informed consent to participate in this study.

## Author Contributions

MB: conceptualization, methodology, software, formal analysis, investigation, writing–original draft. SB-S: investigation, data curation, and writing—review and editing. NM: supervision and writing—review & editing. JN: supervision, writing—review and editing, and funding acquisition. All authors critically revised and made substantial contributions to the final document.

## Funding

This study was partly funded by the Dutch Brain Foundation (grant no. Ps2012-06).

## Conflict of Interest

The authors declare that the research was conducted in the absence of any commercial or financial relationships that could be construed as a potential conflict of interest.

## Publisher's Note

All claims expressed in this article are solely those of the authors and do not necessarily represent those of their affiliated organizations, or those of the publisher, the editors and the reviewers. Any product that may be evaluated in this article, or claim that may be made by its manufacturer, is not guaranteed or endorsed by the publisher.
