# Supplementary material for: Self-Reported Complaints as Prognostic Markers for Outcome After Mild Traumatic Brain Injury in Elderly: A Machine Learning Approach
Source: Front Neurol. 2021 Dec 2;12:751539. doi: 10.3389/fneur.2021.751539 (PMC8674199; doi:10.3389/fneur.2021.751539)
Supplement: Supplementary file 1 [file Data_Sheet_1.DOCX]

Supplementary Material

# APPENDIX

## Preliminary Analysis (patients with mTBI vs. healthy controls)

The purpose of the preliminary analysis was to validate our machine learning-based approach. Therefore, we employed it to identify the subset of self-reported complaints that optimally distinguishes elderly patients with mild traumatic brain injury (mTBI) in the (sub)acute phase after injury from healthy controls (HCs) before performing our main analysis.

## Methods

### *Study population*: Patients with mTBI were recruited as described in the main text (see *Main Text, Methods* section). Additionally, HCs were recruited for the UPFRONT and ReCONNECT studies via social contacts and advertisements, using the same inclusion and exclusion criteria that were described for mTBI patients (see *Main text, Methods* section).

### *Measurements*: The Head Injury Symptoms Checklist (HISC) was used to assess self-reported complaints as described in the main text (see *Main text, Methods* section). For HCs, only the current level of complaints can be measured. Therefore, for this preliminary analysis, only current level of complaints was used to classify mTBI patients and HCs.

### Machine Learning approach

In this preliminary analysis, an SVM-based classification model was used to determine the hyper-plane that maximizes the separating margin between the two classes, namely mTBI patients and HCs (class labels defined as 1 and -1, respectively). Data preparation, model training, backward feature selection procedure, statistical analysis and performance assessment followed the same methodology and algorithms described in the main text (see *Main Text, Methods* section). A few adjustments to the data preparation were performed, if necessary, as described below.

Classification Features. For the classification of mTBI vs. HCs, the features consisted of the severity of each complaint as reported by the participant at the moment of filling out the questionnaire (i.e. not corrected for pre-injury level), ranging from 0 to 2 (0: never, 1: sometimes, 2: often) and thus, k=20.

Training and testing datasets. Data of the UPFRONT and ReCONNECT studies were combined. The cross-validation training and the external validation datasets consisted of a random, stratified selection of 80% of the data for cross-validation training and 20% of the data for external validation using the function “cvpartition” (MATLAB 2020) (see *Main text, Methods* section). For the cross-validation training dataset selection, the number of observations for the minority group was artificially increased using the Synthetic Minority Oversampling Technique (SMOTE) procedure. The dataset for external validation was held out from the cross-validation training process and left unseen until the external validation.

## Results

### Participant characteristics

In total, 214 elderly with mTBI (median age: 67 years, IQR: 9 years) and 47 age-matched healthy controls (median age: 67 years, IQR: 7 years) were included in the preliminary analysis. Independent-samples median tests indicated no significant difference in age between groups (mTBI vs HC).

The prevalence of complaints per mTBI and HC group in the training dataset is presented in Fig. S1.


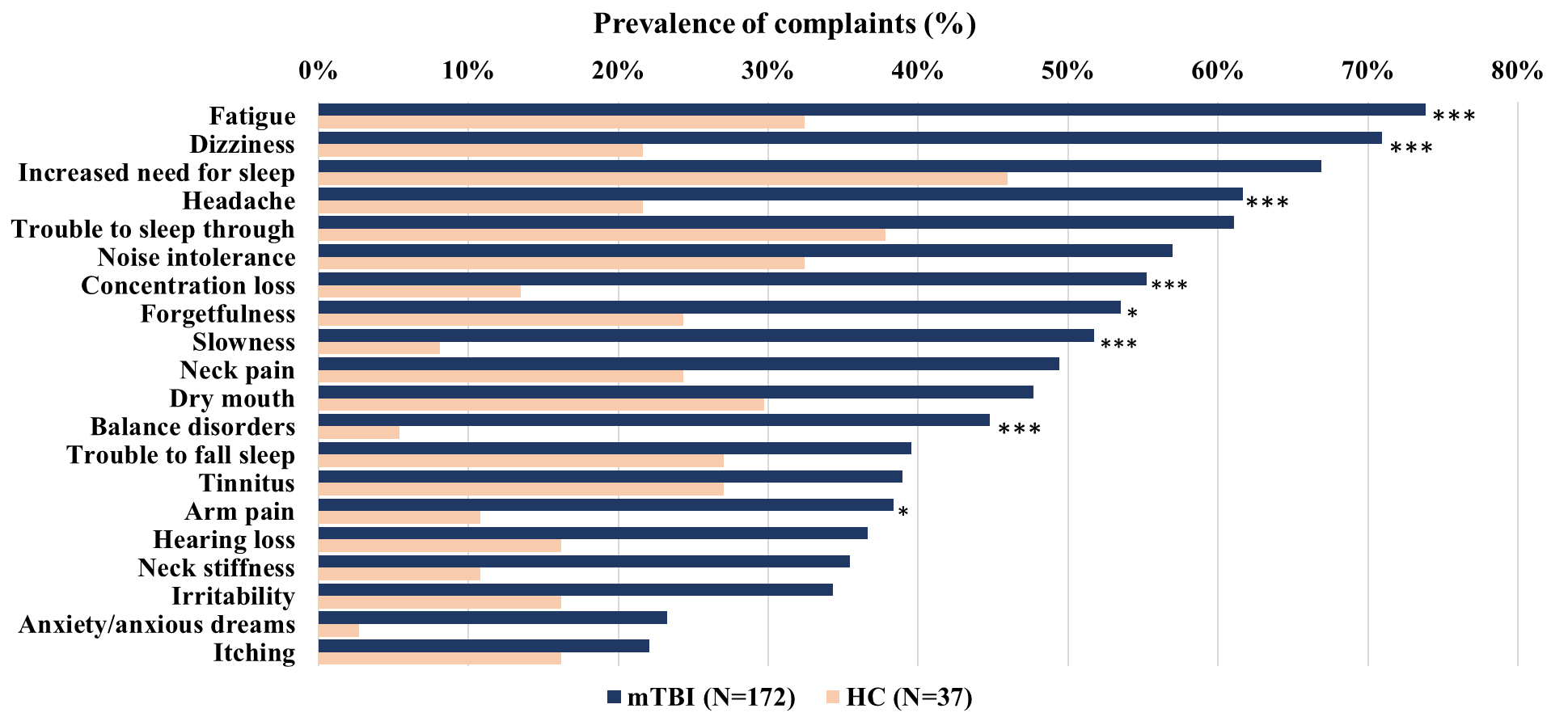


Figure S1– Overview of the prevalence of current complaints (%) for healthy controls and for patients with mTBI two weeks after injury in percentages of the total of each group. Complaints ordered by prevalence in the mTBI group from highest to lowest. Asterisks indicate significant differences between groups (Mann Whitney U-Test, *p<0.05, **p<0.01, ***p<0.001, Bonferroni corrected for multiple comparisons).

### Feature Selection and performance assessment

The ML feature selection procedure identified an optimized classification model containing 9 out of 20 features that achieved the maximum ROC-AUC of 0.91. The selected optimal subset containing 9 features, ranked by order of exclusion, is detailed in Table S1. Dizziness was the last feature to be excluded during the feature selection procedure, although its weight did not reach statistical significance. Three out of the 9 selected features were identified as significant for the classification of mTBI patients vs. HCs: arm pain (Weight: 1.06 [95% CI: 1.02-1.11], p=0.001), balance disorders (Weight: 1.45 [95% CI: 1.34-1.56], p<0.001) and concentration loss (Weight: 0.78 [95% CI: 0.75-0.80], p=0.030), in order of selection (the first feature was the last to be excluded), as shown in Table S1.

Table S1 – Optimal subset of features for classification of patients with mTBI vs. HCs ranked by order of exclusion if selection process continued until the last remaining feature (1: last feature to be excluded). Blue: significant features with positive weight, that contribute to patient with mTBI prediction.

| **Features** | **Selection Ranking** | **Weight, mean, 95% CI [ LL, UL]** ^a^ | **p-value** ^b^ |
| --- | --- | --- | --- |
| **Dizziness** | **1** | 0.53 [0.44, 0.63] | 0.136 |
| **Arm pain** | **2** | 1.06 [1.02, 1.11] | 0.001 |
| **Balance disorders** | **3** | 1.45 [1.34, 1.56] | <0.001 |
| **Fatigue** | **4** | 0.50 [0.45, 0.54] | 0.143 |
| **Neck stiffness** | **5** | 0.50 [0.45, 0.54] | 0.093 |
| **Trouble to sleep through** | **6** | 0.28 [0.23, 0.33] | 0.190 |
| **Anxiety/Anxious dreams** | **7** | 0.71 [0.35, 1.07] | 0.061 |
| **Increased need for sleep** | **8** | -0.30 [-0.34, -0.26] | 0.207 |
| **Concentration loss** | **9** | 0.78 [0.75, 0.80] | 0.030 |

^a^ 95% Confidence interval (CI) values calculated based on 5-fold cross-validation; LL: lower level, UL; upper level. ^b^ Statistical significance based on permutation tests (N models built with random class labels permutations, N=10000)

The final trained, cross-validated model yielded an average ROC-AUC of 0.91[95% CI: 0.84-0.99] (see Fig. S2, A). The confusion matrices displaying the classification results for the cross-validation and for the external validation on an independent testing dataset are shown in Fig. S2 (B and C).


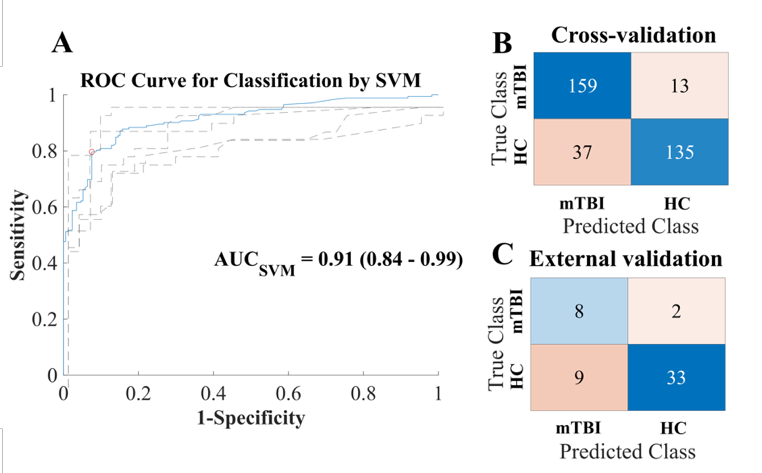


Figure S2– A: Receiver operating characteristic curve for prediction of group (mTBI vs. HC) based on a linear SVM model built with the optimal subselection of discriminating features and 5-fold cross-validation. Dashed lines indicate results per fold. B, C: Confusion matrices for the predictions of the SVM model vs true labels on cross-validation dataset (B) and external validation on independent dataset (C).

Secondary performance measures are detailed in Table S2. The classifier achieved similar performance levels for sensitivity both in the cross-validation (Sensitivity_CV_=0.79 [95%CI 0.74-0.83]) and in the external validation (Sensitivity_EV_=0.79). The performance level for specificity, however, was lower in the external validation (Specificity_EV_=0.80) than in the cross validation (Specificity_CV_=0.93 [95%CI 0.86-0.99]).

Table S2 – Performance of selected SVM classifier on cross-validation dataset and external validation on independent dataset

|  | **HC vs mTBI** | |
| --- | --- | --- |
|  | **Cross-Validation**  Mean, [95% CI] | **External Validation** |
| **Sensitivity** | 0.79 [0.74-0.83] | 0.79 |
| **Specificity** | 0.93 [0.86-0.99] | 0.80 |
| **Total Accuracy** | 0.85 [0.81-0.90] | 0.79 |
| **F1-score** | 0.85 [0.80-.090] | 0.79 |
| **ROC-AUC** | 0.91 [0.84-0.99] | - |

HC: healthy controls; mTBI: mild traumatic brain injury; ROC-AUC: area under the receiver operating characteristic curve.
